# Coumarin-based fluorescent probe for the rapid detection of peroxynitrite ‘AND’ biological thiols†

**DOI:** 10.1039/d0ra02234a

**Published:** 2020-04-03

**Authors:** Luling Wu, Xue Tian, Robin R. Groleau, Jie Wang, Hai-Hao Han, Shaun B. Reeksting, Adam C. Sedgwick, Xiao-Peng He, Steven D. Bull, Tony D. James

**Affiliations:** Department of Chemistry, University of Bath Bath BA2 7AY UK t.d.james@bath.ac.uk lw960@bath.ac.uk; Key Laboratory for Advanced Materials and Joint International Research Laboratory of Precision Chemistry and Molecular Engineering, Feringa Nobel Prize Scientist Joint Research Center, School of Chemistry and Molecular Engineering, East China University of Science and Technology 130 Meilong Rd. Shanghai 200237 China; Materials and Chemical Characterization (MC^2^), University of Bath Bath BA2 7AY UK https://doi.org/10.15125/mx6j-3r54; Department of Chemistry, University of Texas at Austin 105 E 24th Street A5300 Austin TX 78712-1224 USA

## Abstract

A coumarin-based novel ‘AND’ logic fluorescent probe ROS-AHC has been developed for the simultaneous detection of ONOO^−^ and biological thiols. ROS-AHC was shown to exhibit only a very small fluorescence response upon addition of a single GSH or ONOO^−^ analyte. Exposure to both analytes, however, resulted in a significant fluorescence enhancement.

## Abstract 2

A coumarin-based novel ‘AND’ logic fluorescent probe ROS-AHC has been developed for the simultaneous detection of ONOO^−^ and biological thiols.

## Graphical abstract

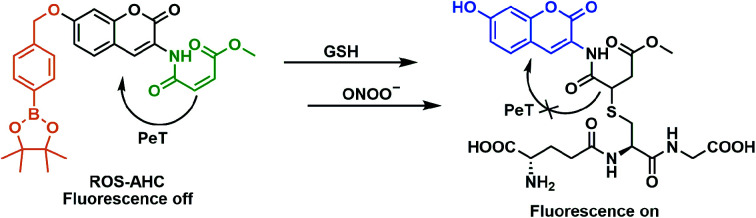

Peroxynitrite (ONOO^−^) is a short-lived reactive oxygen and reactive nitrogen species (ROS and RNS) produced intracellularly by the diffusion-controlled reaction of nitric oxide (NO˙) with superoxide (O_2_˙^−^).^1–3^ Despite playing a key role as a physiological regulator,^4^ it is commonly known for its high reactivity towards most types of biomolecules, causing deleterious effects and irreversible damage to proteins, nucleic acids, and cell membranes.^5,6^ ONOO^−^ is therefore a central biological pathogenic factor in a variety of health conditions such as strokes, reperfusion injuries or inflammatory and neurodegenerative diseases (Parkinson's disease, Alzheimer's disease).^7–9^ Conversely, biothiols such as glutathione and cysteine are endogenous reducing agents, playing a central role in the intracellular antioxidant defence systems.^10–12^ Glutathione (GSH), in particular, is the most abundant biothiol in mammalian cells, and exists as both its reduced GSH form, and as the oxidised disulphide form GSSG.^13–15^ Peroxynitrite and biothiols such as GSH are intimately linked, as abnormal levels of highly oxidising ONOO^−^ can perturb the delicate GSH/GSSG balance, causing irreversible damage to key processes such as mitochondrial respiration.^16^ Thus, abnormal levels of GSH are common in cells undergoing oxidative stress, in which the regulation of and interplay between ONOO^−^ and GSH is closely associated with physiological and pathological processes.^17,18^ One such example is drug-induced liver injury (DILI), in which upregulation of ONOO^−^ occurs in hepatotoxicity. Treatment with GSH could be used to remediate this type of cell injury by depletion of ONOO^−^.^19–22^

One of our core research interests lies in the development of dual analyte chemosensors capable of detecting two distinct analytes such as biological reactive oxygen species and biothiols.^23–26^ Although a wide range of single-analyte probes exist for the detection of ROS and thiols separately,^27–30^ ‘AND’ logic sensors for their simultaneous detection are still rare.^31–33^ We are therefore interested in developing such probes, containing two distinct sensing units, one for each analyte, working simultaneously or in tandem to elicit a fluorescence response.^34^ This approach allows the monitoring of multiple biomolecular events and factors involved in specific disease pathologies, in order to achieve optimal predictive accuracy for diagnosis and prognostication.^35^

Using these principles, our group has recently focused on developing a range of ‘AND’ logic based sensors exploiting a variety of sensing units and mechanisms of fluorescence. Two such probes are shown below: GSH-ABAH (Fig. 1a), an ESIPT probe with a 4-amino-2-(benzo[*d*]thiazol-2-yl)phenol (ABAH) core, employing a maleic anhydride thiol-acceptor group;^31^ and JEG-CAB (Fig. 1b), a coumarin-based probe, this time with a salicylaldehyde homocysteine-reactive unit.^24^ Both of these sensors employ a benzyl boronate ester as their peroxynitrite-reactive unit.

**Fig. 1:**
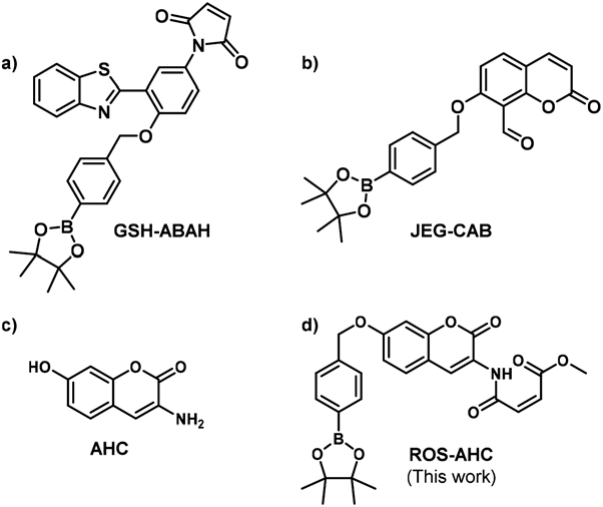
(a) GSH-ABAH, previously reported probe for simultaneous detection of ONOO^−^ and GSH. (b) JEG-CAB, previously reported probe for simultaneous detection of ONOO^−^ and GSH. (c) AHC – a core fluorescent unit that enables the synthesis of ‘AND’ based fluorescent probe for the detection of ONOO^−^ and GSH (d) ROS-AHC, a novel probe detailed in this work for simultaneous detection of ONOO^−^ and GSH.

Herein, we set out to develop an ‘AND’ logic gate based fluorescence probe for simultaneous detection of ONOO^−^ and GSH. 3-Amino-7-hydroxy-2*H*-chromen-2-one (AHC) was chosen as a suitable coumarin fluorophore core for the development of an ‘AND’ logic based sensor, as its free phenol and amine functional groups provided a good opportunity for independent derivatization (Fig. 1).^36–39^

Previous literature reports show that protection of AHC with a maleic anhydride group results in quenching of the coumarin's fluorescence intensity due to photoinduced electron transfer (PeT) processes. This fluorescence is rapidly restored in the presence of biological thiols, however, due to their fast addition to this functional group.^40^ Therefore, we suggested that functionalization of the free phenol of this sensor using a benzyl boronic ester should further block the fluorescence, whilst serving as reporter unit for ONOO^−^. The greatly increased reactivity of peroxynitrite over other ROS towards boronate esters^41–43^ should allow this functionality to act as a peroxynitrite-selective reporter, leading to an ‘AND’ logic based probe for the detection of ONOO^−^ and biological thiols (Fig. 1, Scheme 1). ROS-AHC was synthesized in 5 steps, starting with a 4-step synthesis of compound 1 adapted from literature procedures,^40,44^ followed by the addition of the benzyl boronic pinacol ester (see Scheme S1 ESI†).

**Scheme 1:**
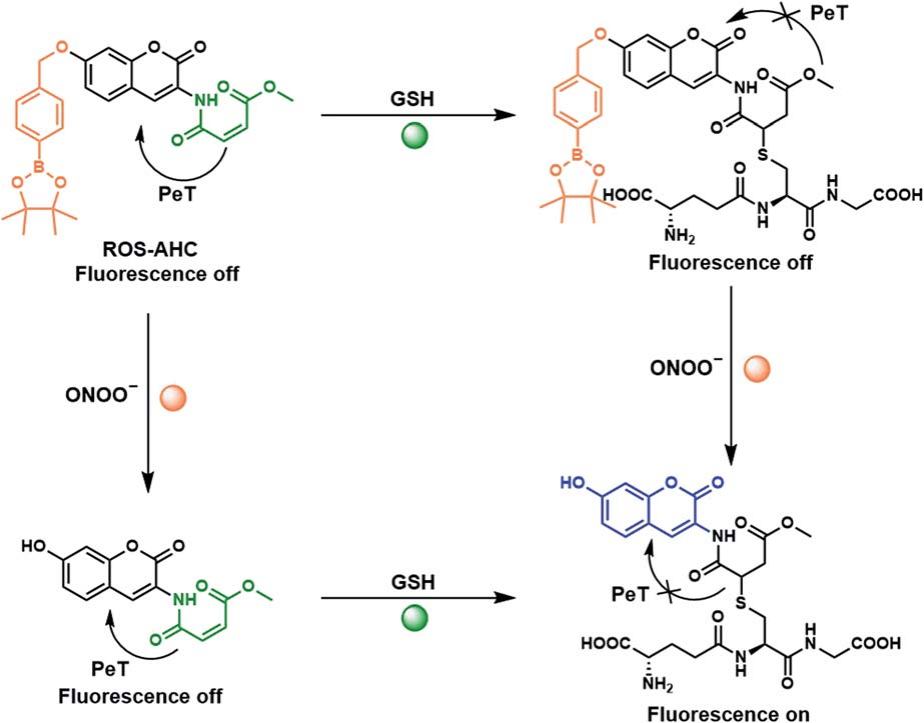
Fluorescence ‘turn on’ mechanism of ROS-AHC in the presence of ONOO^−^ and GSH.

The UV-Vis behaviour of ROS-AHC before and after exposure to both GSH and ONOO^−^ was evaluated in pH 7.40 buffer solution, showing a maximum absorption peak at 340 nm for both the unreacted probe and the probe following exposure to GSH, shifting to 350 nm with the addition of ONOO^−^ to the probe and 365 nm after sequential additions of GSH and ONOO^−^ to the probe (Fig. S1 ESI†). Fluorescence experiments were then carried out. As expected, ROS-AHC was initially non-fluorescent, with a small fluorescence increase upon addition of ONOO^−^ (6 µM) (Fig. 2 and S2 ESI†). Incremental additions of GSH (0–4.5 µM) resulted in a much larger increase in fluorescence intensity (>69-fold, see Fig. 2 and S3 ESI†), demonstrating the need for both GSH and ONOO^−^ in order to achieve a significant ‘turn on’ fluorescence response.

**Fig. 2:**
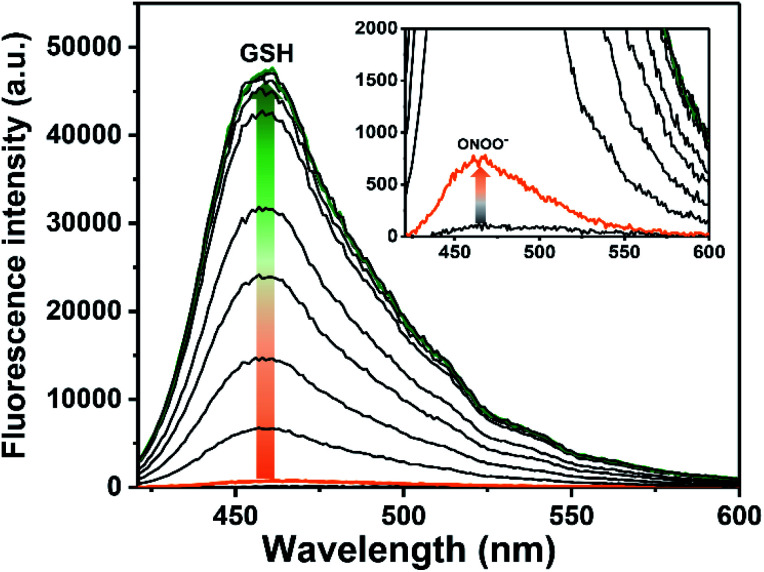
Fluorescence spectra of ROS-AHC (5 µM) with addition of ONOO^−^ (6 µM), wait 5 min then incremental addition of GSH (0–4.5 µM), 5 min incubation before measurements in PBS buffer solution (10 mM, pH = 7.40). Fluorescence intensities were measured with *λ*_ex_ = 400 nm (bandwidth 8 nm). The green line represents the highest intensity after addition of GSH (4 µM).

Similar fluorescence experiments were then carried out in reverse order, with the addition of GSH (6 µM) to ROS-AHC resulting in only a small increase in fluorescence intensity (Fig. 3 and S4 ESI†). As before, incremental addition of the second analyte, in this case ONOO^−^ (0–5.5 µM), resulted in a large increase in fluorescence intensity (>46-fold, Fig. 3 and S5 ESI†), confirming that ROS-AHC requires both GSH and ONOO^−^ for a full fluorescence ‘turn on’ response.

**Fig. 3:**
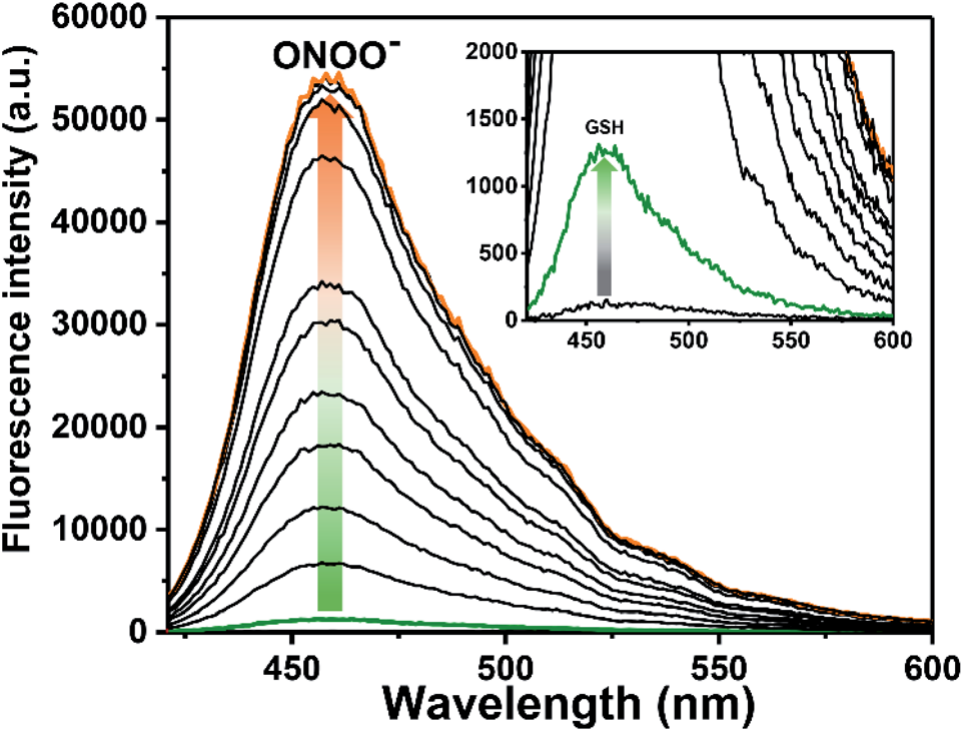
Fluorescence spectra of ROS-AHC (5 µM) with addition of GSH (6 µM), wait 5 min then incremental addition of ONOO^−^ (0–5.5 µM) with 5 min incubation before measurements in PBS buffer solution (10 mM, pH = 7.40). Fluorescence intensities were measured with *λ*_ex_ = 400 nm (bandwidth 8 nm). The orange line shows the highest intensity after addition of ONOO^−^ (5 µM).

Subsequently, the selectivity of this probe towards both analytes was evaluated. A range of amino acids were evaluated (Fig. S6 ESI†), with only thiol-containing analytes (glutathione, cysteine and homocysteine) eliciting significant fluorescence response, whilst non-thiol amino acids led to no changes in fluorescence intensity. A broad screen of ROS analytes was also carried out, demonstrating excellent selectivity for ONOO^−^, even over H_2_O_2_ (Fig. S7 ESI†).

The time-dependent response of ROS-AHC with both ONOO^−^ and GSH was also examined (Fig. S8 and S9 ESI†). After initial addition of GSH or ONOO^−^ to the probe, subsequent addition of the second analyte triggered a rapid and significant increase in fluorescence, achieving maximum fluorescence intensity within 78 s in both cases. Furthermore, LC-MS experiments confirmed the formation of the suggested non-fluorescent intermediates, as well as the final fluorescent species shown in Scheme 1 (Fig S10, S11 and S12†).

In summary, we have developed a coumarin-based dual-analyte ‘AND’ logic fluorescent sensor, ROS-AHC, for the simultaneous detection of ONOO^−^ and biological thiols. ROS-AHC has shown high sensitivity and selectivity towards both ONOO^−^ and biological thiols.

## Conflicts of interest

No conflicts of interest.

## Supplementary Material

RA-010-D0RA02234A-s001

## References

[cit1] Szabó C., Ischiropoulos H., Radi R. (2007). Nat. Rev. Drug Discovery.

[cit2] Pacher P., Beckman J. S., Liaudet L. (2007). Physiol. Rev..

[cit3] Trujillo M., Radi R. (2002). Arch. Biochem. Biophys..

[cit4] Ferdinandy P. (2006). Br. J. Pharmacol..

[cit5] Storz G., Imlayt J. A. (1999). Curr. Opin. Microbiol..

[cit6] Ascenzi P., di Masi A., Sciorati C., Clementi E. (2010). BioFactors.

[cit7] Sarchielli P., Galli F., Floridi A., Floridi A., Gallai V. (2003). Amino Acids.

[cit8] Ischiropoulos H., Beckman J. S. (2003). J. Clin. Invest..

[cit9] Wink D. A., Vodovotz Y., Laval J., Laval F., Dewhirst M. W., Mitchell J. B. (1998). Carcinogenesis.

[cit10] Stamler J. S., Slivka A. (1996). Nutr. Rev..

[cit11] Iwata S., Hori T., Sato N., Ueda-Taniguchi Y., Yamabe T., Nakamura H., Masutani H., Yodoi J. (1994). J. Immunol..

[cit12] Nakamura H., Nakamura K., Yodoi J. (1997). Annu. Rev. Immunol..

[cit13] Dringen R., Gutterer J. M., Hirrlinger J. (2000). Eur. J. Biochem..

[cit14] Morgan B., Ezeriņa D., Amoako T. N. E., Riemer J., Seedorf M., Dick T. P. (2013). Nat. Chem. Biol..

[cit15] Lim C. S., Masanta G., Kim H. J., Han J. H., Kim H. M., Cho B. R. (2011). J. Am. Chem. Soc..

[cit16] Virág L., Szabó É., Gergely P., Szabó C. (2003). Toxicol. Lett..

[cit17] Marshall K.-A., Reist M., Jenner P., Halliwell B. (1999). Free Radical Biol. Med..

[cit18] Bolaños J. P., Heales S. J. R., Land J. M., Clark J. B. (1995). J. Neurochem..

[cit19] Shuhendler A. J., Pu K., Cui L., Uetrecht J. P., Rao J. (2014). Nat. Biotechnol..

[cit20] Li D., Wang S., Lei Z., Sun C., El-Toni A. M., Alhoshan M. S., Fan Y., Zhang F. (2019). Anal. Chem..

[cit21] Jiang W.-L., Li Y., Wang W.-X., Zhao Y.-T., Fei J., Li C.-Y. (2019). Chem. Commun..

[cit22] Kelly G. S. (1998). Altern. Med. Rev..

[cit23] Wu L., Sedgwick A. C., Sun X., Bull S. D., He X.-P., James T. D. (2019). Acc. Chem. Res..

[cit24] Wu L., Gardiner J. E., Kumawat L. K., Han H.-H., Guo R., Li X., He X.-P., Elmes R. B. P., Sedgwick A. C., Bull S. D., James T. D. (2019). RSC Adv..

[cit25] Wu L., Liu L., Han H.-H., Tian X., Odyniec M. L., Feng L., Sedgwick A. C., He X.-P., Bull S. D., James T. D. (2019). New J. Chem..

[cit26] Weber M., Mackenzie A. B., Bull S. D., James T. D. (2018). Anal. Chem..

[cit27] Sedgwick A. C., Gardiner J. E., Kim G., Yevglevskis M., Lloyd M. D., Jenkins A. T. A., Bull S. D., Yoon J., James T. D. (2018). Chem. Commun..

[cit28] Wu L., Yang Q., Liu L., Sedgwick A. C., Cresswell A. J., Bull S. D., Huang C., James T. D. (2018). Chem. Commun..

[cit29] Odyniec M. L., Han H.-H., Gardiner J. E., Sedgwick A. C., He X.-P., Bull S. D., James T. D. (2019). Front. Chem..

[cit30] Wu L., Wang Y., Weber M., Liu L., Sedgwick A. C., Bull S. D., Huang C., James T. D. (2018). Chem. Commun..

[cit31] Wu L., Han H.-H., Liu L., Gardiner J. E., Sedgwick A. C., Huang C., Bull S. D., He X.-P., James T. D. (2018). Chem. Commun..

[cit32] Sedgwick A. C., Han H.-H., Gardiner J. E., Bull S. D., He X.-P., James T. D. (2018). Chem. Sci..

[cit33] Yu F., Li P., Li G., Zhao G., Chu T., Han K. (2011). J. Am. Chem. Soc..

[cit34] Kolanowski J. L., Liu F., New E. J. (2018). Chem. Soc. Rev..

[cit35] Romieu A. (2015). Org. Biomol. Chem..

[cit36] Zhang H., Zhang C., Liu R., Yi L., Sun H. (2015). Chem. Commun..

[cit37] Ang C. Y., Tan S. Y., Lu Y., Bai L., Li M., Li P., Zhang Q., Selvan S. T., Zhao Y. (2014). Sci. Rep..

[cit38] García-Beltrán O., Cassels B. K., Pérez C., Mena N., Núñez M. T., Martínez N. P., Pavez P., Aliaga M. E. (2014). Sensors.

[cit39] Nicosia C., Cabanas-Danés J., Jonkheijm P., Huskens J. (2012). ChemBioChem.

[cit40] Yi L., Li H., Sun L., Liu L., Zhang C., Xi Z. (2009). Angew. Chem., Int. Ed..

[cit41] Palanisamy S., Wu P.-Y., Wu S.-C., Chen Y.-J., Tzou S.-C., Wang C.-H., Chen C.-Y., Wang Y.-M. (2017). Biosens. Bioelectron..

[cit42] Sun X., Xu Q., Kim G., Flower S. E., Lowe J. P., Yoon J., Fossey J. S., Qian X., Bull S. D., James T. D. (2014). Chem. Sci..

[cit43] Sikora A., Zielonka J., Lopez M., Joseph J., Kalyanaraman B. (2009). Free Radical Biol. Med..

[cit44] Jiang J., Gou C., Luo J., Yi C., Liu X. (2012). Inorg. Chem. Commun..

