# Supplementary material for: Coumarin-based fluorescent probe for the rapid detection of peroxynitrite ‘AND’ biological thiols
Source: RSC Adv. 2020 Apr 3;10(23):13496–9. doi: 10.1039/d0ra02234a (PMC9051425; doi:10.1039/d0ra02234a)
Supplement: RA-010-D0RA02234A-s001 [file RA-010-D0RA02234A-s001.pdf]

Electronic Supplementary Material (ESI) for RSC Advances.
This journal is © The Royal Society of Chemistry 2021

Coumarin-based fluorescent probe for the rapid detection of peroxynitrite 'AND' biological thiols

Luling Wu,[a‡*] Xue Tian,[a‡] Robin R. Groleau,[a] Jie Wang,[b] Hai-Hao Han,[b] Shaun B. Reeksting,[c] Adam C. Sedgwick,[d] Xiao-Peng He,[b] Steven D. Bull,[a] and Tony D. James[a*]

[a.] Department of Chemistry, University of Bath, Bath, BA2 7AY, United Kingdom.

[b.] Key Laboratory for Advanced Materials and Joint International Research Laboratory of Precision Chemistry and Molecular Engineering, Feringa Nobel Prize Scientist Joint Research Center, School of Chemistry and Molecular Engineering, East China University of Science and Technology, 130 Meilong Rd., Shanghai 200237, China.

[c.] Materials and Chemical Characterization (MC^2), University of Bath, Bath BA2 7AY, United Kingdom (https://doi.org/10.15125/mx6j-3r54).

[d.] Department of Chemistry, University of Texas at Austin, 105 E 24th street A5300, Austin, TX 78712-1224, USA.

‡Equal contribution

E-mail: t.d.james@bath.ac.uk; lw960@bath.ac.uk

UPDATED 1st February 2021: The original version of this ESI was first published on 3rd April 2020. This version of the ESI replaces the previous copy in which there was a minor error in the NMR characterization data for Compound 3, and Figure S15 was incorrectly shown.

Table of contents

1. **UV-Vis and fluorescence analysis S-2**
2. **Generation of various ROS S-10**
3. **Mass spectroscopic analysis S-11**
4. **Synthesis and characterisation of compounds 1-4 and ROS-AHC S-14**
5. **NMR spectra S-16**
6. **Author contributions S-21**
7. **References S-21**

1. UV-Vis and fluorescence analysis

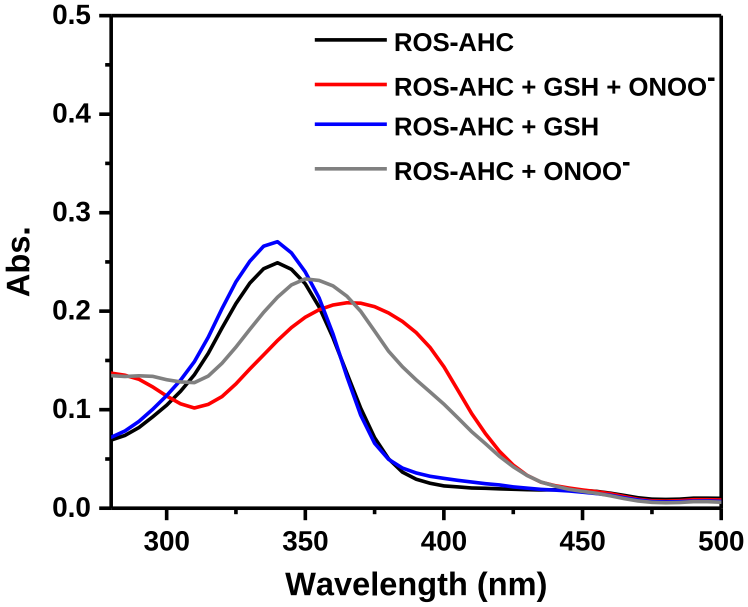

Figure S1. Absorption spectra of **ROS-AHC** (20 μM) only with and without $ONOO^-$ (18 μM) wait 5 min/ GSH (26 μM) wait 5 min, and with addition of GSH (26 μM) wait 5 min then addition of $ONOO^-$ (18 μM) with 5 min incubation before measurements in PBS buffer solution (10 mM, pH = 7.40).

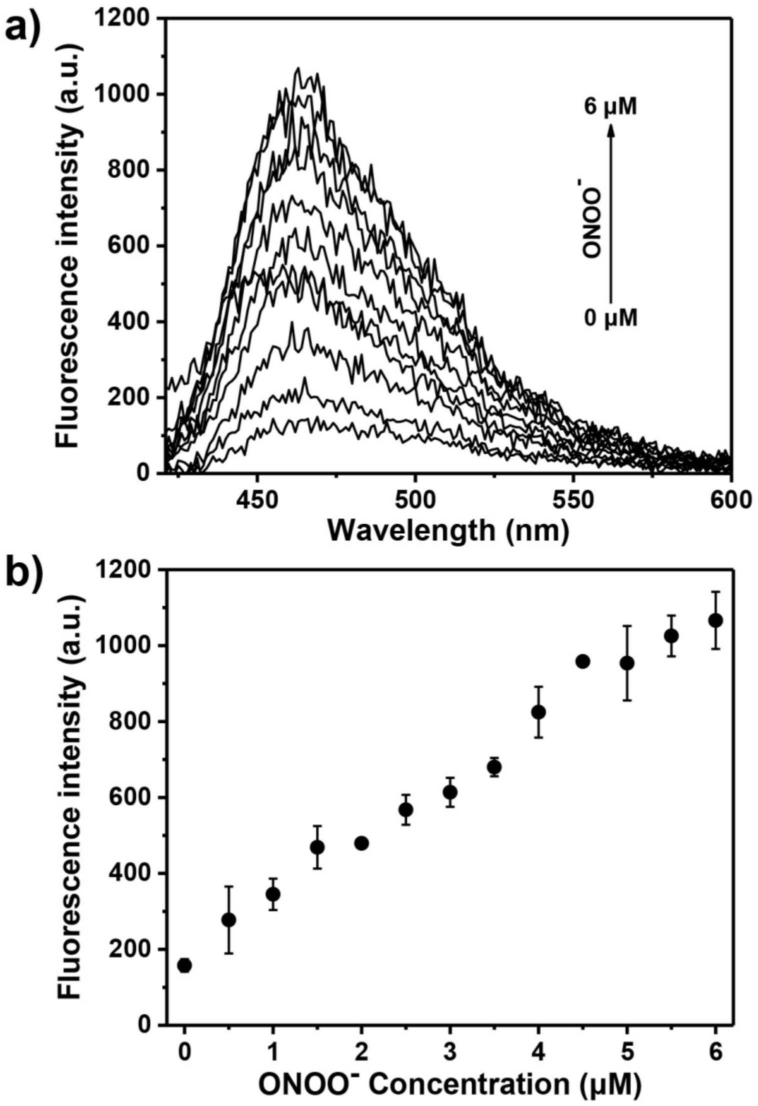

Figure S2. (a) Fluorescence spectra of **ROS-AHC** (5 μM) with increasing additions of $ONOO^-$ (from 0 to 6 μM) in PBS buffer solution (10 mM, pH = 7.40) after 5 min. (b) Fluorescence intensity changes (based on the intensities at 461 nm) against $ONOO^-$ concentration. λ_{ex} = 400 nm (bandwidth 8 nm).

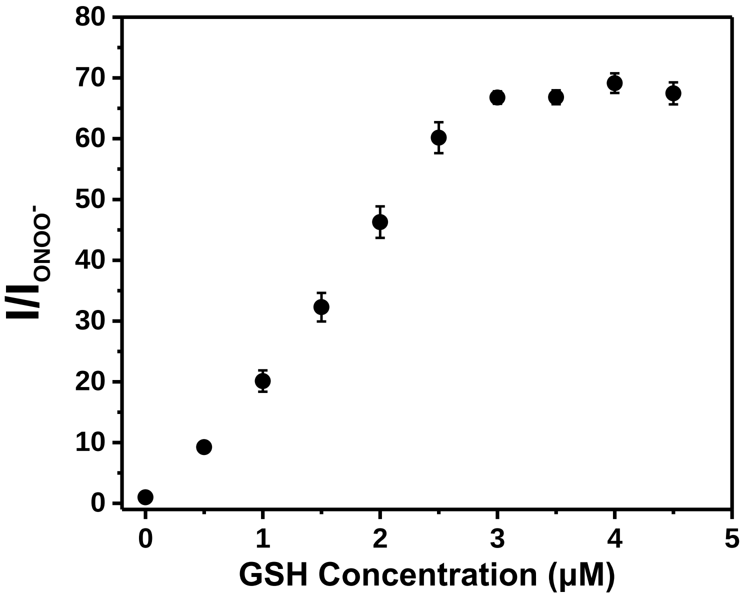

Figure S3. Fluorescence intensity changes (I/I_{ONOO^-}) for **ROS-AHC** (5 μM) with addition of $ONOO^-$ (6 μM), wait 5 min, then additions of GSH (0 – 4.5 μM) with 5 min incubation before measurement in PBS buffer solution (10 mM, pH = 7.40). λ_{ex} = 400 nm (bandwidth 8 nm)/ λ_{em} = 461 nm.

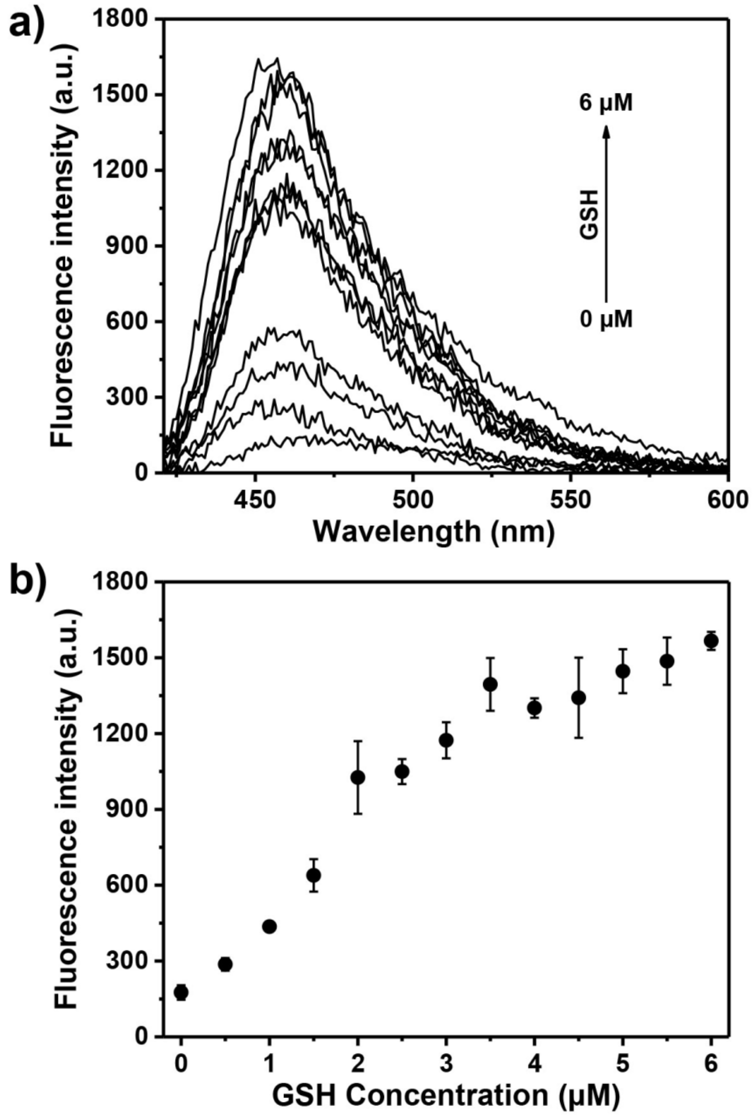

Figure S4. (a) Fluorescence spectra of **ROS-AHC** (5 μM) with increasing additions of GSH (from 0 to 6 μM) with 5 min incubation before measurement in PBS buffer solution (10 mM, pH = 7.40). (b) Fluorescence intensity changes (based on the intensities at 461 nm) against GSH concentration. λ_{ex} = 400 nm (bandwidth 8 nm).

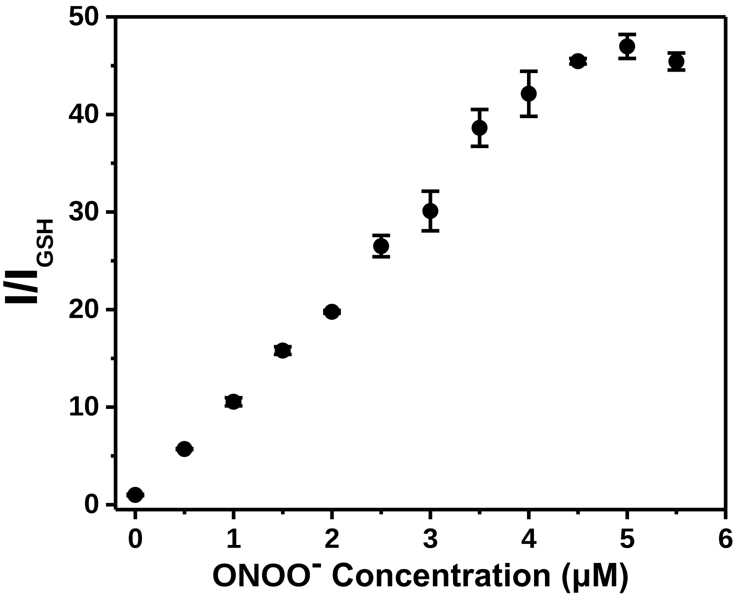

Figure S5. Fluorescence intensity changes (I/I_{GSH}) for **ROS-AHC** (5 µM) with addition of GSH (6 µM), wait 5 min, then additions of $ONOO^-$ (0 – 5.5 µM) with 5 min incubation before measurement in PBS buffer solution (10 mM, pH = 7.40). λ_{ex} = 400 nm (bandwidth 8 nm)/ λ_{em} = 461 nm.

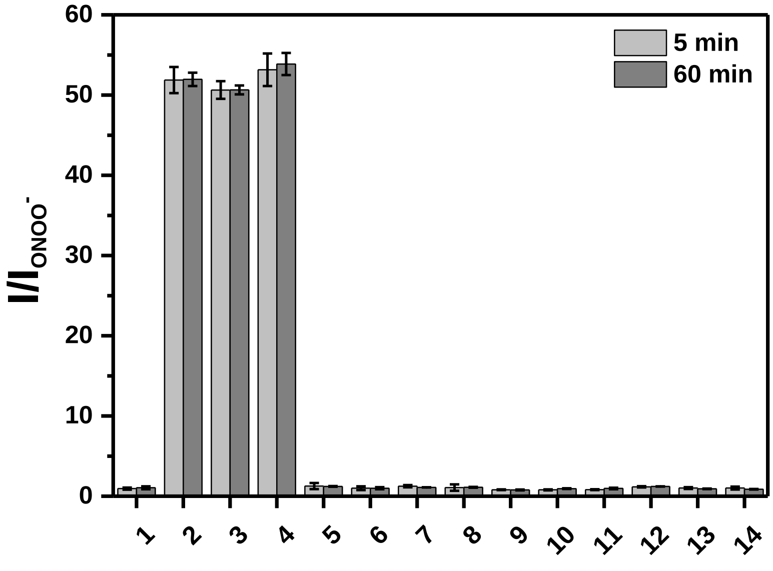

Figure S6. Selectivity bar chart of **ROS-AHC** (5 µM) with addition of $ONOO^-$ (6 µM), wait 5 min, then addition of various amino acids wait 5 min or 60 min before measurement in PBS buffer solution (10 mM, pH = 7.40), 1 – blank; 2 – GSH (Glutathione, 4 µM); 3 – Cys (Cysteine, 4 µM); 4 – Hcy (Homocysteine, 4 µM); 5 – Glu (Glutamic acid, 500 µM); 6 – Phe (Phenylalanine, 500 µM); 7 – Asp (Aspartic acid, 500 µM); 8 – Pro (Proline, 500 µM); 9 – Val

(Valine, 500 μM); 10 – Ser (Serine, 500 μM); 11 – Lys (Lysine, 500 μM); 12 – Iso (Isoleucine, 500 μM); 13 – His (Histidine, 500 μM); 14 – Arg (Argnine, 500 μM). λ_{ex} = 400 nm (bandwidth 8 nm)/ λ_{em} = 461 nm.

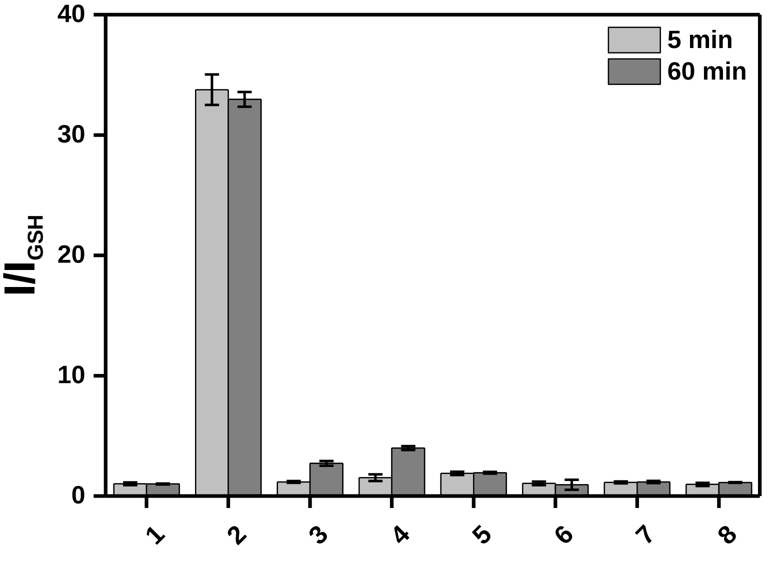

Figure S7. Selectivity bar chart of **ROS-AHC** (5 μM) with addition of GSH (6 μM), wait 5 min, then addition of various ROS wait 5 min or 60 min before measurement in PBS buffer solution (10 mM, pH = 7.40). 1 – blank; 2 – $ONOO^-$ (5 μM); 3 – HOCl (100 μM); 4 – H_2O_2 (100 μM); 5 – ROO• (100 μM); 6 – •OH (100 μM); 7 – $O_2^{\bullet-}$ (100 μM); 8 – $^1O_2$ (100 μM). λ_{ex} = 400 nm (bandwidth 8 nm)/ λ_{em} = 461 nm.

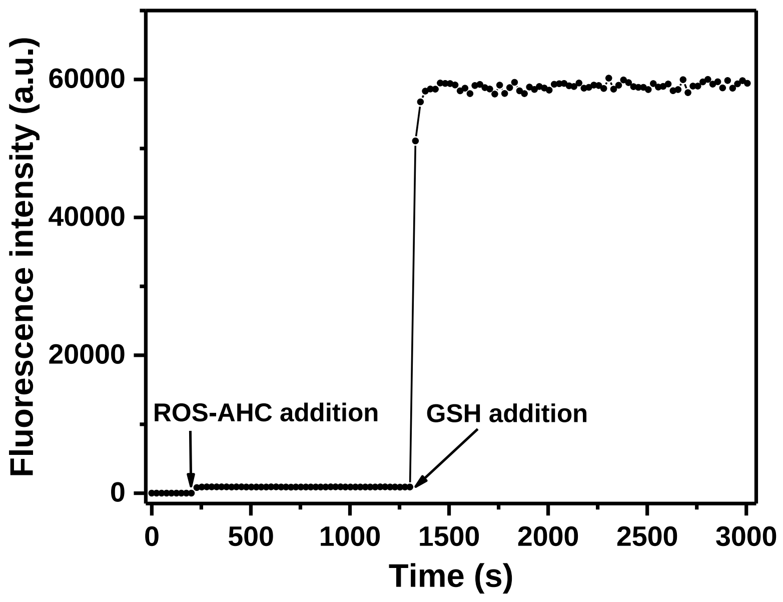

Figure S8. Fluorescence intensity over time of the addition of **ROS-AHC** (5 μM) followed by the addition of GSH (4 μM) to $ONOO^-$ (6 μM) in PBS buffer solution (10 mM, pH = 7.40). λ_{ex} = 400 nm (bandwidth 8 nm)/ λ_{em} = 461 nm.

Experimental for Figure S8: A solution of $ONOO^-$ (6 μM) in PBS buffer solution was placed in a Greiner Bio-One microplate (96-well, PS, f-bottom (chimney well), black-walled), and the intensity was measured every 25 s for 200 s. A solution of **ROS-AHC** (6 μM) was then pumped into this solution, and fluorescence was then measured every 25 s from 228 s to 1303 s. A solution of GSH (4 μM) was then added, and fluorescence was measured every 25 s from 1331 s to 3006 s.

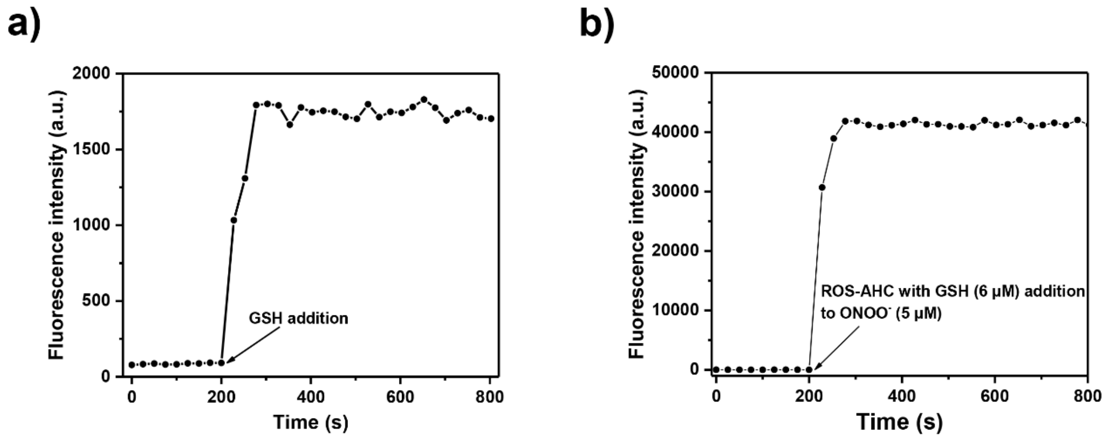

Figure S9. (a) Fluorescence intensity over time of the addition of **ROS-AHC** (5 μM) with addition of GSH (6 μM) at 200 s in PBS buffer solution (10 mM, pH = 7.40). λ_{ex} = 400 nm (bandwidth 8 nm)/ λ_{em} = 461 nm. (b) Fluorescence intensity over time of the addition of the addition of a pre-mixed (5 min) solution of **ROS-AHC** (5 μM) and GSH (6 μM) to $ONOO^-$ (5 μM) at 200 s in PBS buffer solution. λ_{ex} = 400 nm (bandwidth 8 nm)/ λ_{em} = 461 nm.

Experimental for Figure S9: (a) A solution of **ROS-AHC** (5 μM) in PBS buffer solution was placed in a Greiner Bio-One microplate (96-well, PS, f-bottom (chimney well), black-walled), and the intensity was measured every 25 s for 200 s. A solution of GSH (6 μM) was then pumped into this solution, and fluorescence was measured every 25 s from 228 s to 803 s. (b) A solution of $ONOO^-$ (6 μM) in PBS buffer solution was place in a Greiner Bio-One microplate (96-well, PS, f-bottom (chimney well), black-walled), and the intensity was measured every 25 s for 200 s. A solution of **ROS-AHC** (5 μM) and GSH (6 μM), premixed for 5 min, was then pumped into this solution, and the fluorescence was measured every 25 s from 228 s to 803 s.

2. Generation of various ROS

ROO•

ROO• was generated from 2, 2'-azobis (2-amidinopropane) dihydrochloride. AAPH (2, 2' azobis (2-amidinopropane) dihydrochloride, 1 M) was added into deionizer water, and then stirred at 37 °C for 30 min.

$O_2^{•-}$

Superoxide was generated from KO_2. KO_2 and 18-crown-6 ether (2.5 eq) were dissolved in DMSO to afford a 0.25 M solution.

•OH

Hydroxyl radical was generated by the Fenton reaction. To prepare •OH solution, hydrogen peroxide (H_2O_2, 10 eq) was added to $Fe(ClO_4)_2$ in deionised water.

$^1O_2$

$^1O_2$ was generated by reacting H_2O_2 (1 mM) with NaClO (1 mM). The solution of H_2O_2 was added in one portion to the aqueous solution of NaClO and stir for 2 minutes, using the prepared solution immediately.

$ONOO^-$

0.6 M $NaNO_2$, 0.6 M HC1, 0.7 M H_2O_2 was added simultaneously to a 3 M NaOH solution at 0 ºC. The concentration of peroxynitrite in a 0.5 M NaOH aqueous solution was determined from the absorption at 302 nm (Ɛ = 1670 M^{-1} cm^{-1}).

ClO^-

The concentration of ClO^- was determined from the absorption at 292 nm (Ɛ = 350 M^{-1} cm^{-1}).

H_2O_2

The concentration of H_2O_2 was determined from the absorption at 240 nm (Ɛ = 43.6 M^{-1} cm^{-1}).

3. Mass spectroscopic analysis

Compound Table

Compound Label	RT (min)	Observed mass (m/z)	Neutral observed mass (Da)	Theoretical mass (Da)	Mass error (ppm)	Isotope match score (%)
Cpd 1: C27 H28 B N O8	0.68	506.1990	504.1948	504.1944	0.78	98.86

Mass errors of between -5.00 and 5.00 ppm with isotope match scores above 60% are considered confirmation of molecular formulae

Figure: Extracted ion chromatogram (EIC) of compound.

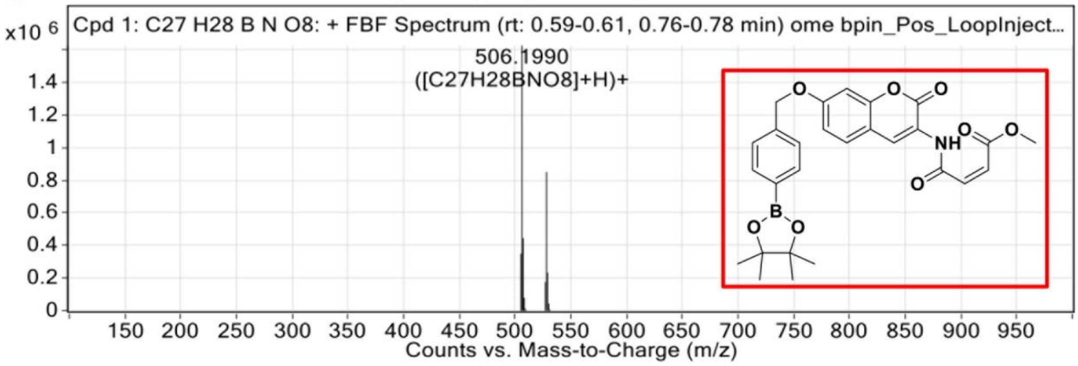

Figure: Full range view of Compound spectra and potential adducts.

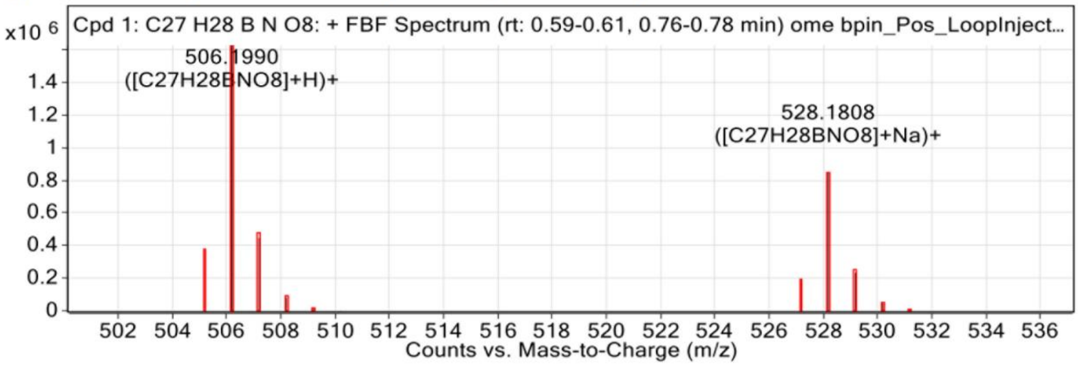

Figure S10. HRMS spectrum of **ROS-AHC** (45 μM).

Compound Table

Compound Label	RT (min)	Observed mass (m/z)	Neutral observed mass (Da)	Theoretical mass (Da)	Mass error (ppm)	Isotope match score (%)
Cpd 1: C14 H11 N O6	4.15	290.0657	289.0586	289.0586	-0.25	74.23

Mass errors of between -5.00 and 5.00 ppm with isotope match scores above 60% are considered confirmation of molecular formulae

Figure: Extracted ion chromatogram (EIC) of compound.

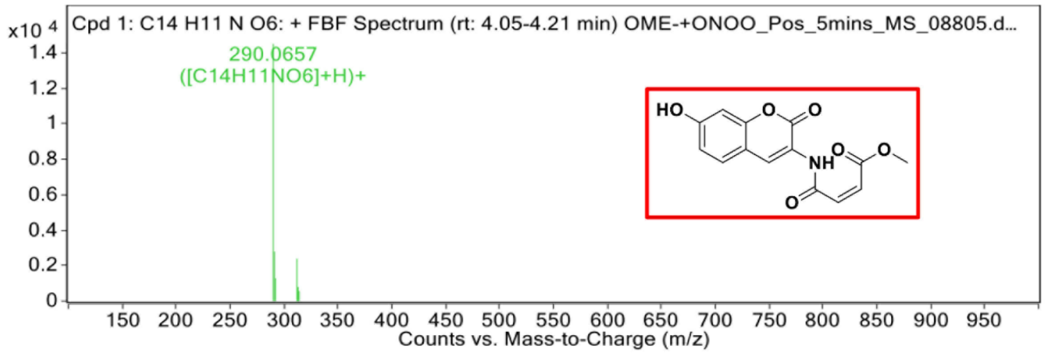

Figure: Full range view of Compound spectra and potential adducts.

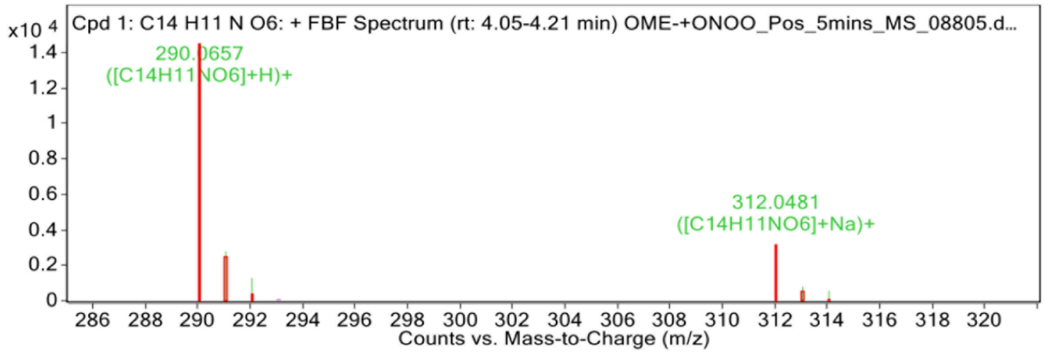

Figure S11. LC-MS spectrum of **ROS-AHC** (45 μM) + ONOO$^-$ (1.5 equiv.).

Compound Table

Compound Label	RT (min)	Observed mass (m/z)	Neutral observed mass (Da)	Theoretical mass (Da)	Mass error (ppm)	Isotope match score (%)
Cpd 1: C24 H28 N4 O12 S	0.96	597.1491	596.1404	596.1424	-3.47	72.99

Mass errors of between -5.00 and 5.00 ppm with isotope match scores above 60% are considered confirmation of molecular formulae

Figure: Extracted ion chromatogram (EIC) of compound.

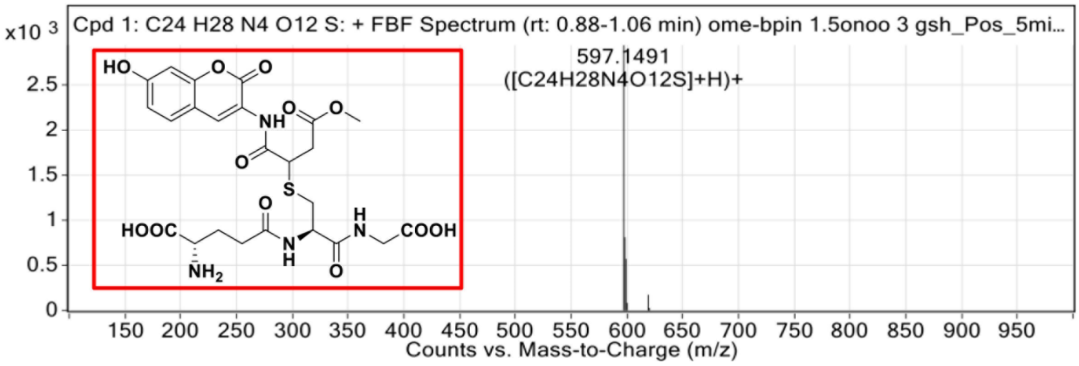

Figure: Full range view of Compound spectra and potential adducts.

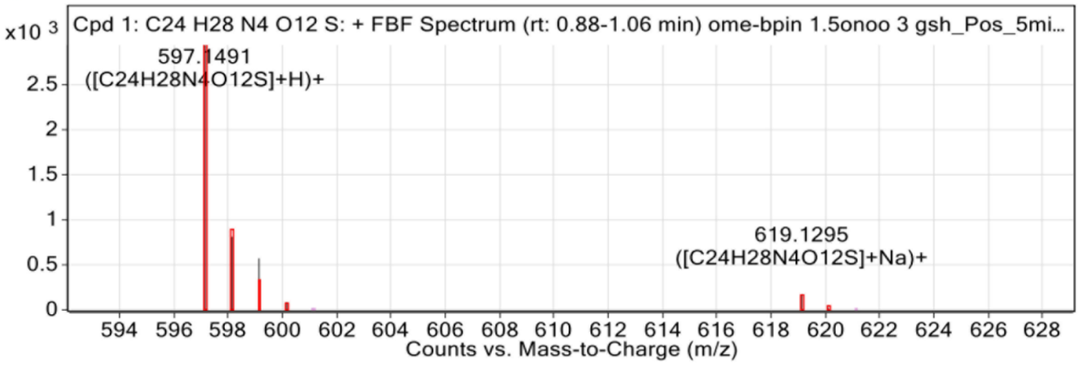

Figure S12. LC-MS spectrum of **ROS-AHC** (45 μM) + $ONOO^-$ (1.5 equiv.) + GSH (3.0 equiv.).

4. Synthesis and characterisation of compounds 1-4 and ROS-AHC

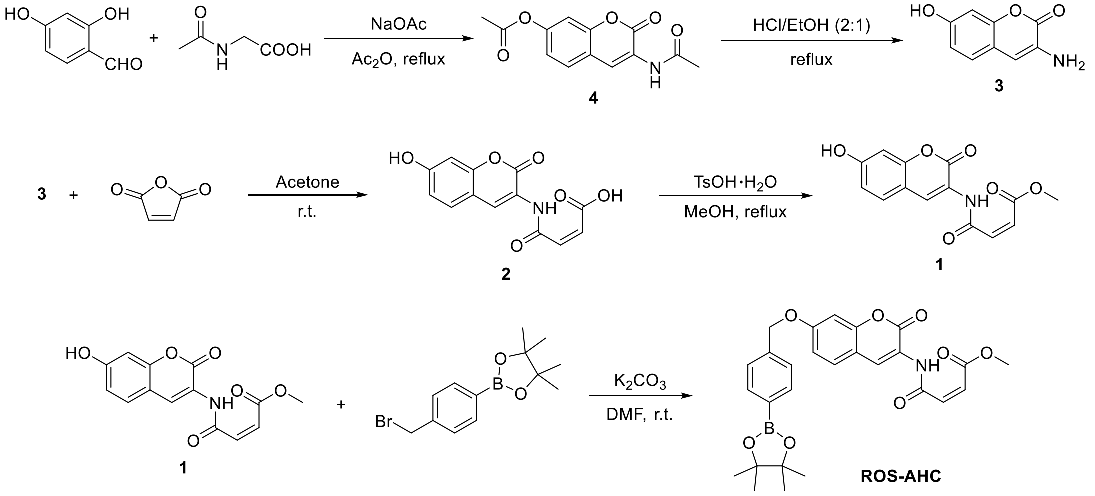

Scheme S1. Synthesis of target **ROS-AHC**.

Synthesis of compounds 1-4

Compounds **1-4** were synthesized using adapted literature procedures.[1,2]

Compound 4

M.p. 241 °C; ^{1}H NMR (500 MHz, DMSO-d_6) δ_H 9.75 (s, 1H), 8.62 (s, 1H), 7.74 (d, J = 8.5 Hz, 1H), 7.27 (d, J = 2.1 Hz, 1H), 7.13 (dd, J = 8.5, 2.2 Hz, 1H), 2.30 (s, 3H), 2.17 (s, 3H); ^{13}C NMR (126 MHz, DMSO-d_6) δ_C 170.2 (s), 168.9 (s), 157.3 (s), 150.9 (s), 149.9 (s), 128.5 (s), 124.1 (s), 123.1 (s), 119.0 (s), 117.4 (s), 109.7 (s), 23.9 (s), 20.8 (s). HRMS (ESI$^+$): calc. for $C_{13}H_{11}NO_5$ $[M+H]^+$ 262.0710 *m/z*, found 262.0711 *m/z*.

Compound 3

M.p. 247 °C; ^{1}H NMR (500 MHz, DMSO-d_6) δ_H 9.80 (s, 1H), 7.23 (d, J = 8.4 Hz, 1H), 6.69 – 6.65 (m, 3H), 5.22 (bs, 2H); ^{13}C NMR (126 MHz, DMSO-d_6) δ_C 159.0 (s), 156.1 (s), 149.3 (s), 130.2 (s), 125.7 (s), 113.5 (s), 112.9 (s), 109.8 (s), 101.8 (s). HRMS (ESI$^+$): calc. for $C_9H_7NO_3$ $[M+H]^+$ 178.0499 *m/z*, found 178.0500 *m/z*.

Compound 2

M.p. 247 °C; ^{1}H NMR (500 MHz, DMSO-d_6) ^{1}H NMR (500 MHz, DMSO-d_6) δ_H 12.96 (bs, 1H), 10.41 (s, 1H), 10.14 (s, 1H), 8.61 (s, 1H), 7.55 (d, J = 8.5 Hz, 1H), 6.80 (dd, J = 8.5, 2.3 Hz, 1H), 6.74 (d, J = 2.2 Hz, 1H), 6.62 (d, J = 12.1 Hz, 1H), 6.40 (d, J = 12.0 Hz, 1H); ^{13}C NMR (126 MHz, DMSO-d_6) δ_C 167.6 (s), 163.6 (s), 159.8 (s), 157.7 (s), 151.7 (s), 132.2 (s), 129.2 (s), 128.7 (s), 126.6 (s), 120.6 (s), 113.7 (s), 111.3 (s), 102.0 (s). HRMS (ESI$^+$): calc. for $C_{13}H_9NO_6$ $[M+H]^+$ 276.0503 *m/z*, found 276.0502 *m/z*.

Compound 1

M.p. 189 °C; ^{1}H NMR (500 MHz, DMSO-d_6) δ_H 10.45 (bs, 1H), 10.05 (s, 1H), 8.57 (s, 1H), 7.56 (d, J = 8.6 Hz, 1H), 6.81 (dd, J = 8.5, 2.3 Hz, 1H), 6.75 (d, J = 2.2 Hz, 1H), 6.72 (d, J = 11.8 Hz, 1H), 6.50 (d, J = 11.8 Hz, 1H), 3.69 (s, 3H); ^{13}C NMR (126 MHz, DMSO-d_6) δ_C 167.1

(s), 163.1 (s), 159.8 (s), 157.7 (s), 151.7 (s), 130.9 (s), 129.5 (s), 129.2 (s), 126.6 (s), 120.6 (s), 113.7 (s), 111.3 (s), 101.9 (s), 51.6 (s). HRMS (ESI$^+$): calc. for $C_{14}H_{11}NO_6$ $[M+Na]^+$ 312.0479 *m/z*, found 312.0485 *m/z*.

Synthesis of ROS-AHC

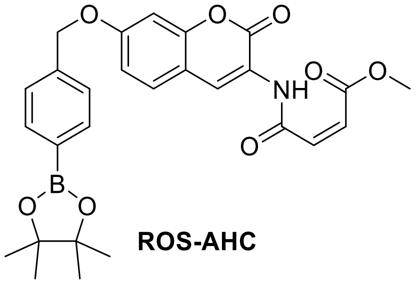

ROS-AHC

4-Bromomethylphenylboronic acid pinacol ester (0.22 g, 0.74 mmol) and K_2CO_3 (0.10 g, 0.74 mmol) were added to a solution of **1** (0.18 g, 0.62 mmol) in dry DMF (6 mL) under a N_2 atmosphere. The resulting suspension was stirred at room temperature for 5 h. The reaction mixture was diluted with EtOAc (90 mL) and washed with brine (90 mL × 3), dried over anhydrous Na_2SO_4 and concentrated *in vacuo*. The crude product obtained was purified by flash chromatography (SiO_2, 30% EtOAc in petroleum ether) to afford the desired product **ROS-AHC** as a yellow solid (72 mg, 23% yield). M.p. 177 °C; ^{1}H NMR (500 MHz, $CDCl_3$) δ_H 9.95 (s, 1H), 8.76 (s, 1H), 7.84 (d, J = 8.0 Hz, 2H), 7.43 (d, J = 8.0 Hz, 2H), 7.40 (d, J = 8.6 Hz, 1H), 6.95 (dd, J = 8.7, 2.4 Hz, 1H), 6.89 (d, J = 2.2 Hz, 1H), 6.42 (d, J = 12.7 Hz, 1H), 6.27 (d, J = 12.7 Hz, 1H), 5.14 (s, 2H), 3.86 (s, 3H), 1.35 (s, 12H); ^{13}C NMR (126 MHz, DMSO-d_6) δ_C 166.3 (s), 163.1 (s), 160.6 (s), 158.8 (s), 151.9 (s), 139.1 (s), 136.3 (s), 135.3 (s), 129.0 (s), 127.1 (s), 126.7 (s), 125.6 (s), 121.8 (s), 114.0 (s), 113.4 (s), 102.0 (s), 84.0 (s), 70.6 (s), 52.9 (s), 25.0 (s). HRMS (ESI$^+$): calc. for $C_{27}H_{28}BNO_8$ $[M+H]^+$ 506.1986 *m/z*, found 506.1990 *m/z*.

5. NMR spectra

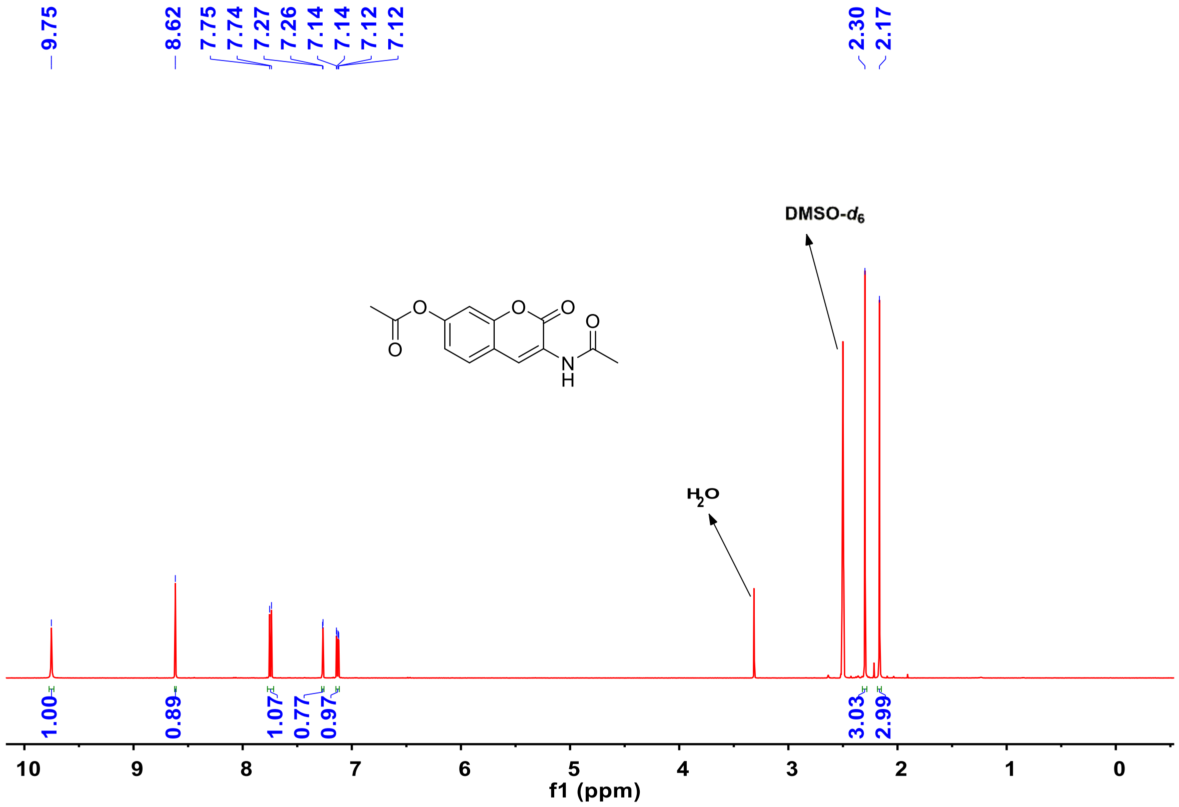

Figure S13. ^{1}H NMR (500 MHz, DMSO – d_6) of compound **4**.

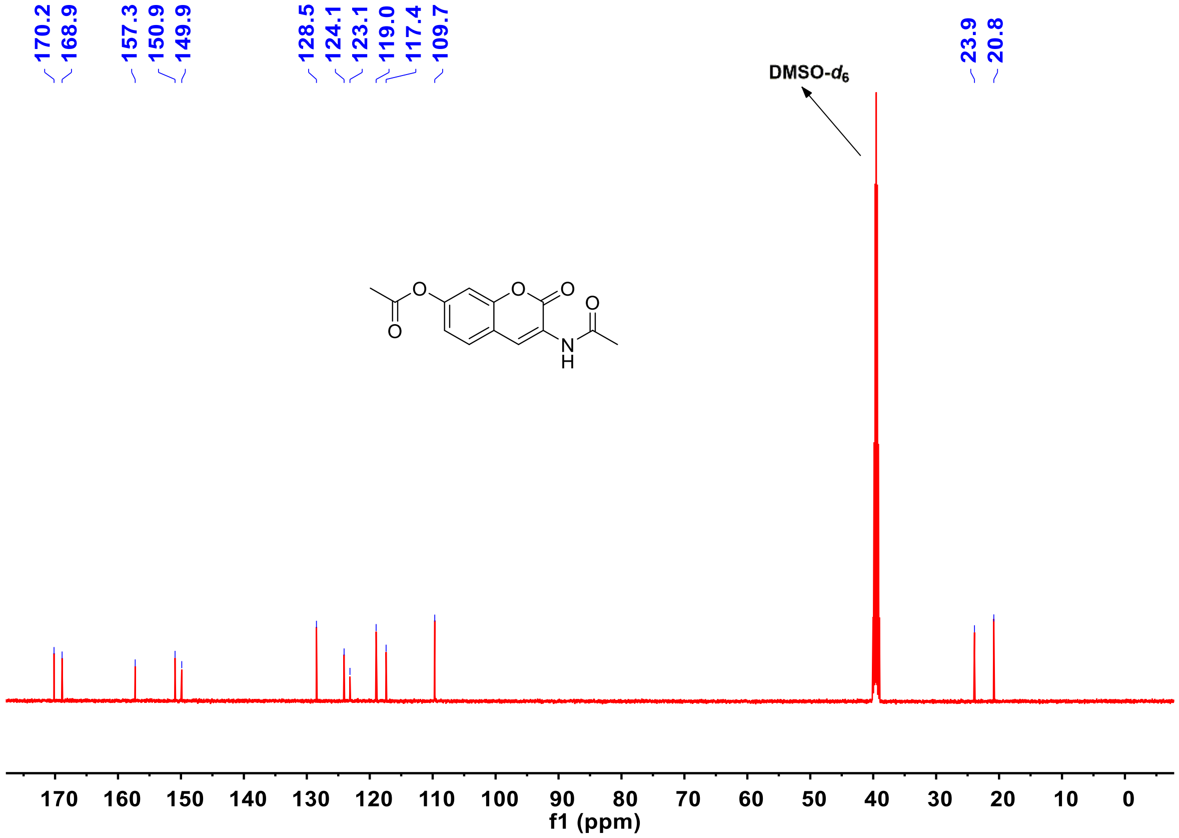

Figure S14. ^{13}C{^{1}H} NMR (126 MHz, DMSO – d_6) of compound **4**.

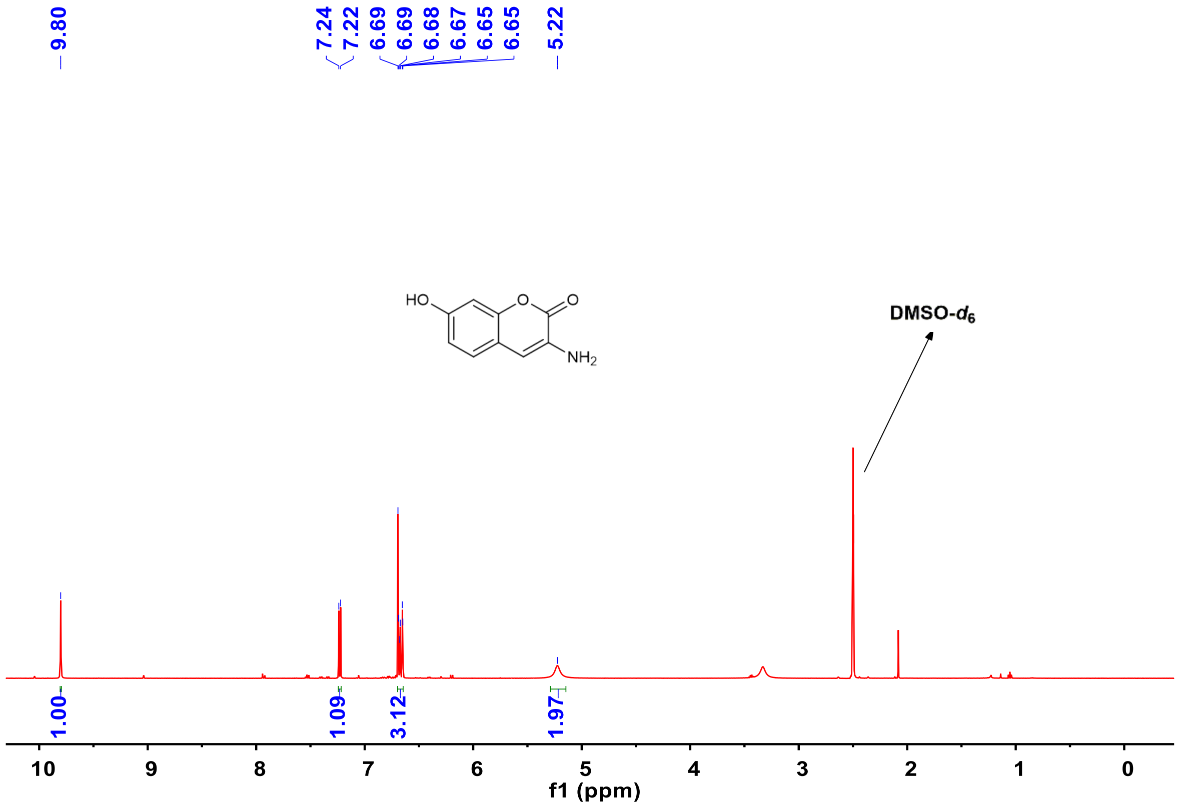

Figure S15. ^{1}H NMR (500 MHz, DMSO – d_6) of compound **3**.

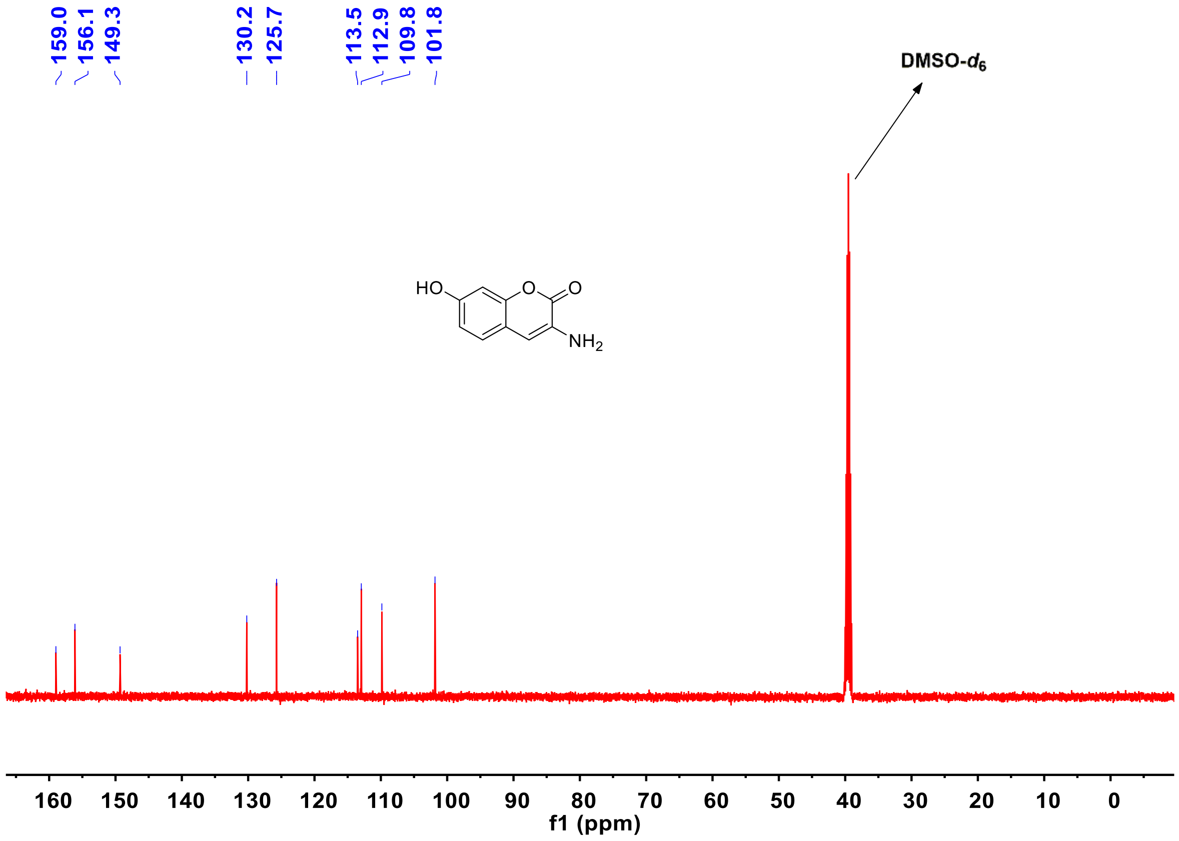

Figure S16. $^{13}C\{^{1}H\}$ NMR (126 MHz, DMSO – d_6) of compound **3**.

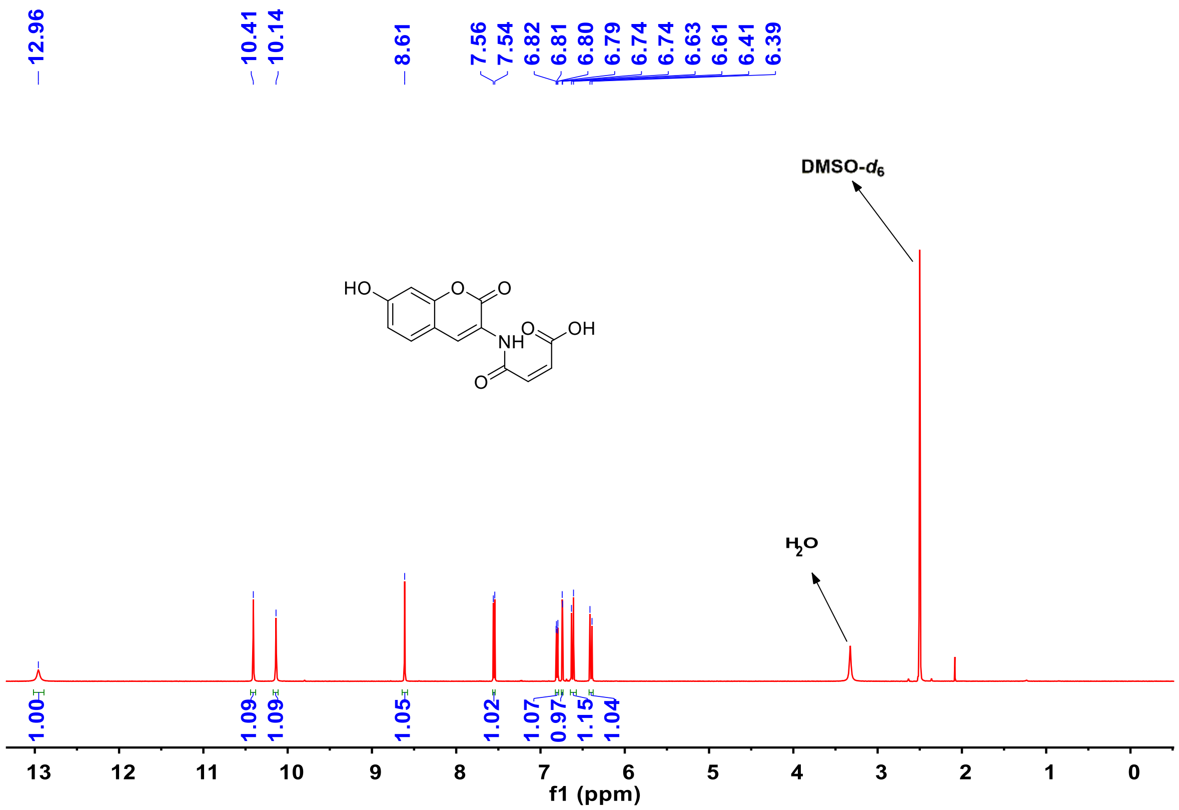

Figure S17. ^{1}H NMR (500 MHz, DMSO – d_6) of compound **2**.

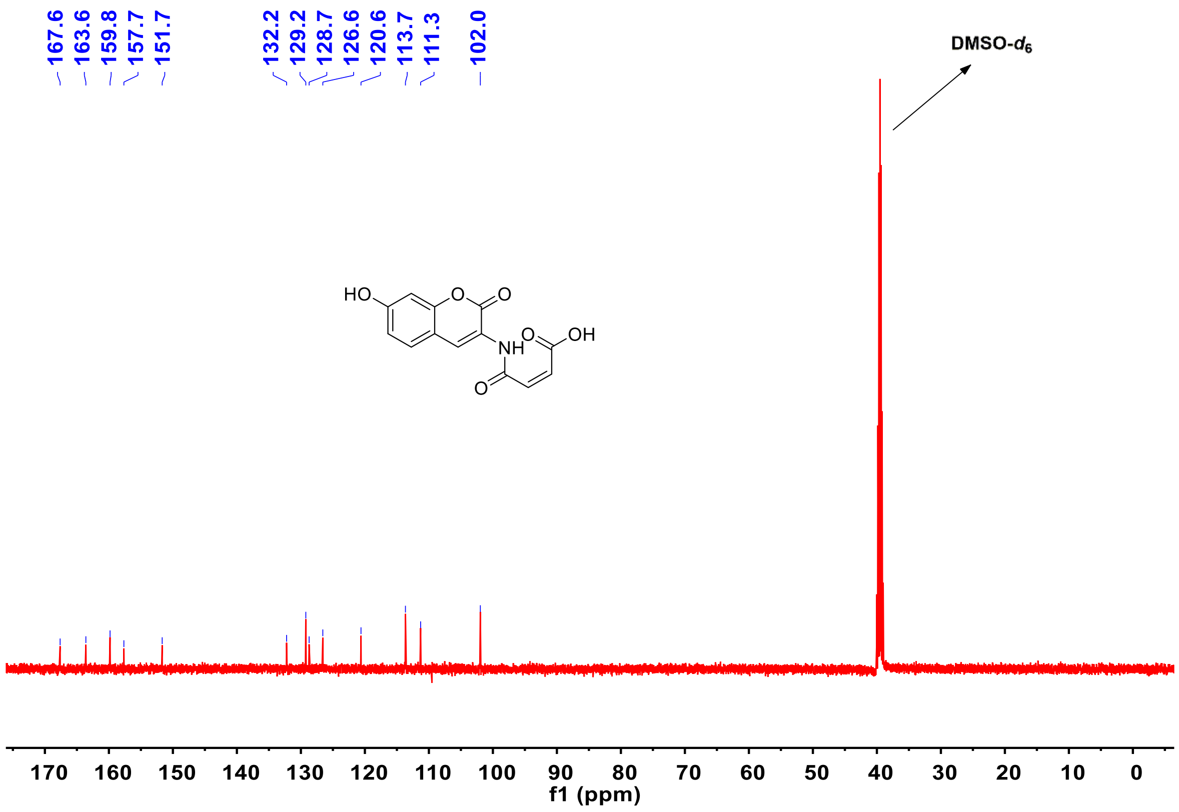

Figure S18. ^{13}C{^{1}H} NMR (126 MHz, DMSO – d_6) of compound **2**.

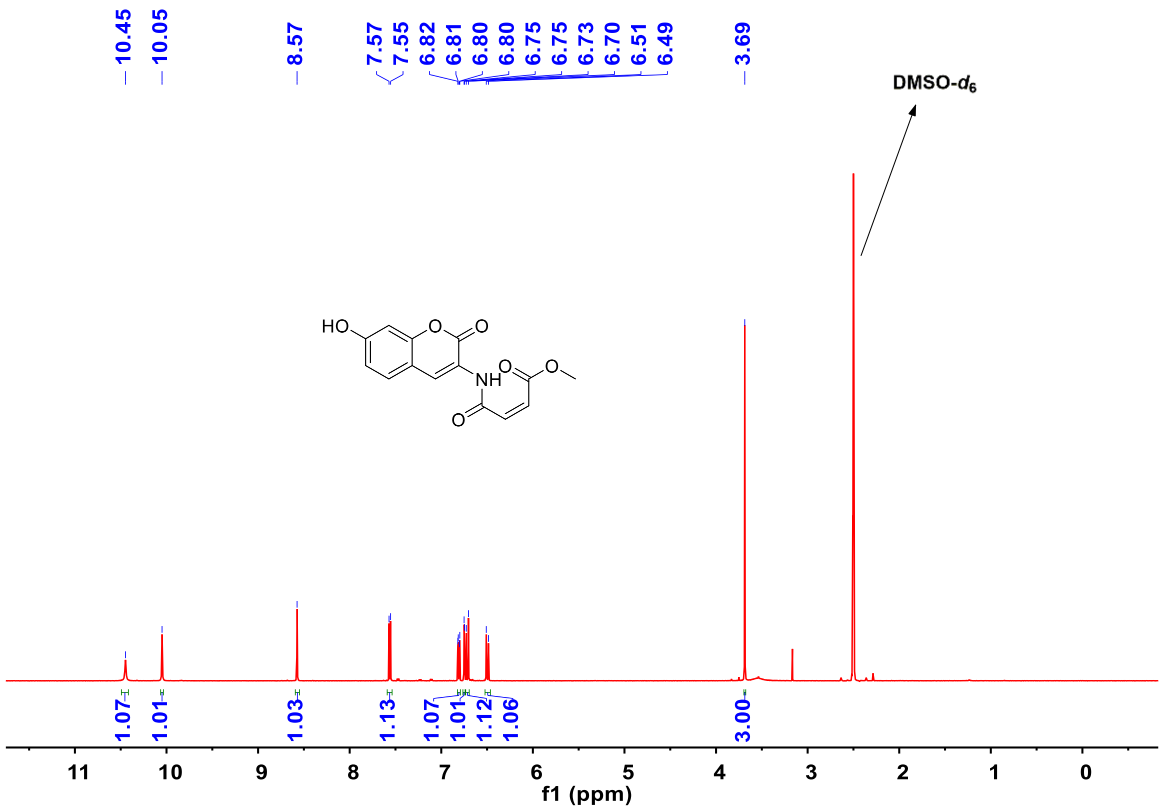

Figure S19. ^{1}H NMR (500 MHz, DMSO – d_6) of compound **1**.

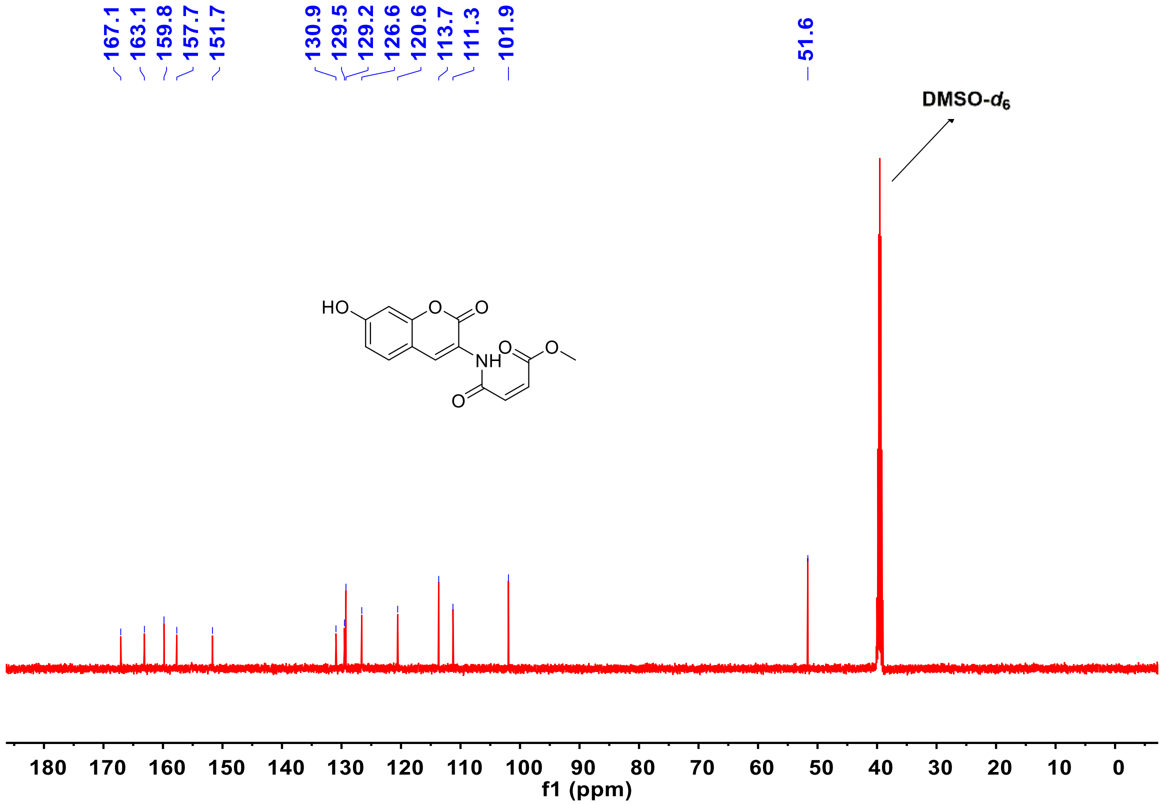

Figure S20. $^{13}C\{^1H\}$ NMR (126 MHz, DMSO – d_6) of compound **1**.

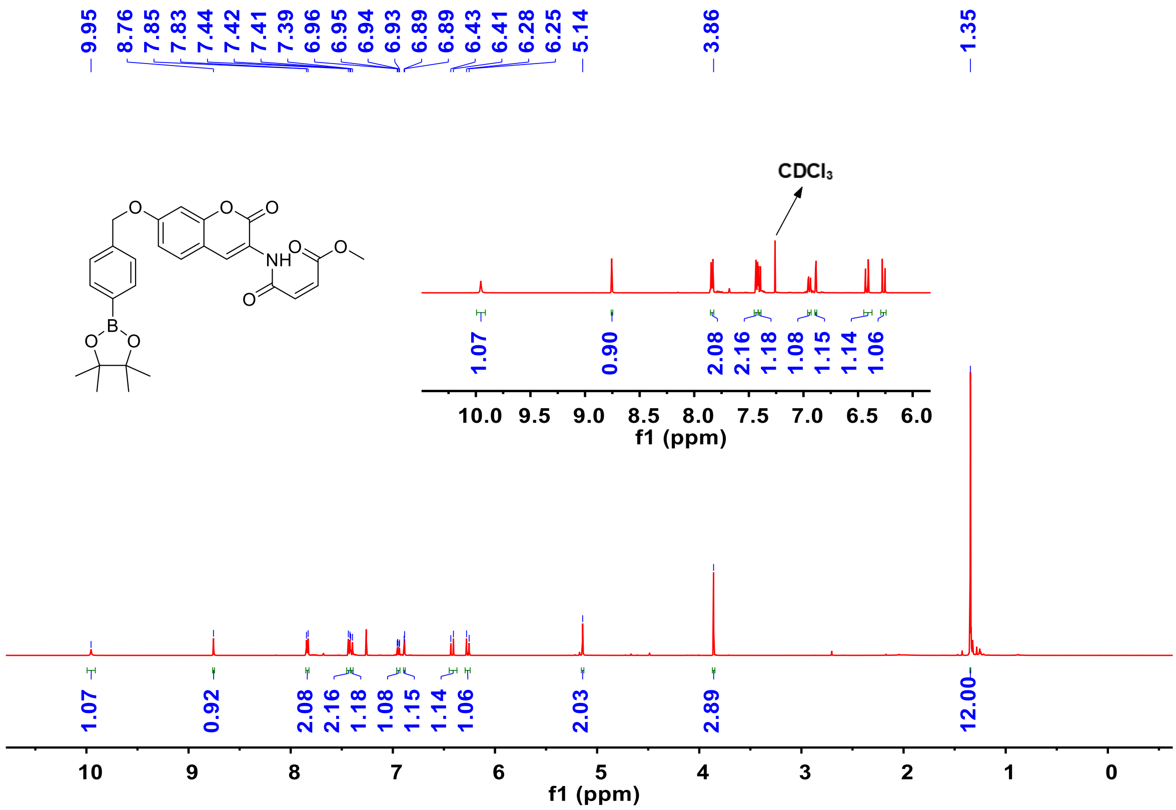

Figure S21. ^{1}H NMR (500 MHz, $CDCl_3$) of **ROS-AHC**.

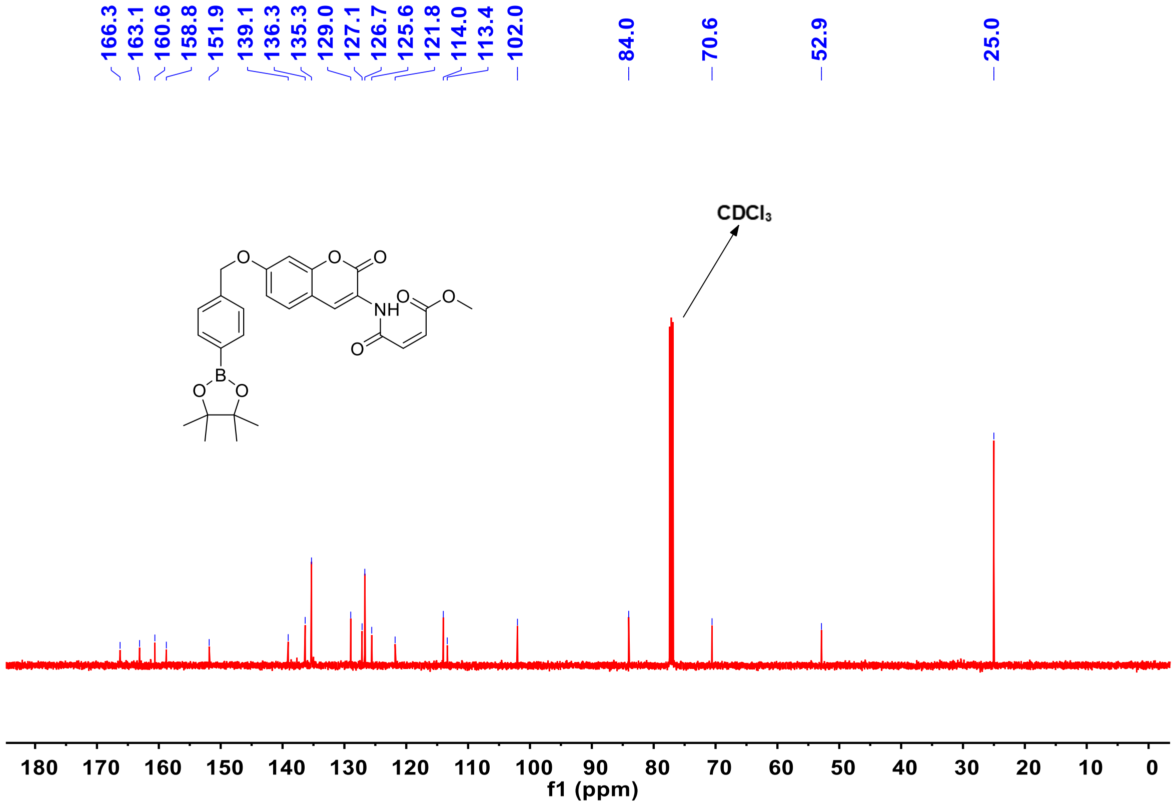

Figure S22. $^{13}C\{^{1}H\}$ NMR (126 MHz, $CDCl_3$) of **ROS-AHC**.

6. Author contributions

Luling Wu – conceived the idea, synthesized the probe and wrote the manuscript.
Xue Tian – wrote the manuscript with Luling Wu and carried out the optical experiments
Robin R. Groleau – provided advice and reviewed and edited the manuscript
Jie Wang – aided Hai-Hao Han with the cellular experiments
Hai-Hao Han – carried out the cellular experiments
Shaun B. Reeksting – helped with the mass spectroscopic analysis
Adam C. Sedgwick – provided advice and reviewed and edited the manuscript
Xiao-Peng He – supervisor of Hai-Hao Han and Jie Wang
Steven D. Bull – supervisor of Luling Wu, Xue Tian and Robin R. Groleau
Tony D. James – lead supervisor

7. References

1 J. Jiang, C. Gou, J. Luo, C. Yi and X. Liu, *Inorg. Chem. Commun.*, 2012, **15**, 12–15.

2 L. Yi, H. Li, L. Sun, L. Liu, C. Zhang and Z. Xi, *Angew. Chem., Int. Ed.*, 2009, **48**, 4034–4037.
